# Cell factories that manufacture microvesicles containing gene silencing RNA prodrugs

**DOI:** 10.1093/pnasnexus/pgag121

**Published:** 2026-04-17

**Authors:** Yanan Feng, Weijing Xu, Ning Deng, Jing Jin, Xiaohong Tang, Jaishree Garhyan, Stanley N Cohen

**Affiliations:** Department of Genetics, Stanford University School of Medicine, Stanford, CA 94305, USA; Department of Genetics, Stanford University School of Medicine, Stanford, CA 94305, USA; Department of Genetics, Stanford University School of Medicine, Stanford, CA 94305, USA; Vitalant Research Institute, San Francisco, CA 94105, USA; Department of Genetics, Stanford University School of Medicine, Stanford, CA 94305, USA; In Vitro Biosafety Level 3 (BSL3) Service Center, Stanford University School of Medicine, Stanford, CA 94305, USA; Department of Genetics, Stanford University School of Medicine, Stanford, CA 94305, USA; Department of Medicine, Stanford University School of Medicine, Stanford, CA 94305, USA

**Keywords:** antisense, shRNA, ARMMs, extracellular vesicles, SARS-CoV-2

## Abstract

Among the vehicles investigated as delivery agents for antisense RNAs are extracellular vesicles (EVs) released from cultured cells. Arrestin domain–containing 1 (ARRDC1)-mediated microvesicles (ARMMs) are naturally occurring EVs that uniquely are formed by outward budding of cytoplasmic membranes. Previous work has shown that ARMMs production is quantitatively controlled by the ARRDC1 protein and that ARRDC1 and macromolecules attached to it are loaded into nascent ARMMs during ARMM formation. Here, we report a strategy for constructing cell factories that biologically manufacture both ARMMs and short hairpin RNA (shRNA)-like antisense RNA precursors, designated as shT-RNAs. Human HEK293T cells mutated in the endoribonuclease DICER1 were engineered to express a fusion protein containing components of ARRDC1 and the trans-activator of transcription (Tat) peptide encoded by the HIV-1 virus. Prodrug RNAs were constructed by replacing the canonical loop regions of shRNAs with a 24-nucleotide segment derived from the Tat-binding trans-activation response (TAR) element. We show that a truncated TAR sequence embedded within the structural framework of shT-RNAs enables their linkage to the ARRDC1–Tat fusion protein and consequent loading of the RNAs into nascent ARMMs, and that RNA is protected by ARMMs membranes from attack by external ribonucleases. We further show that prodrug modules consisting of shT-RNAs that target different genomic sequences of SARS-CoV-2 virus and are manufactured as components of a single transcript can, when delivered by ARMMs, be activated to suppress virus RNA production. Our results indicate that the ARMMs-based platform we have designed can deliver gene-silencing RNAs in the form of biologically produced shRNA-like prodrugs.

Significance statement Nearly six decades ago, it was discovered that complementary nucleic acid sequences introduced into mammalian cells can selectively interfere with gene expression in a reversible and highly specific manner. Although antisense RNA-mediated gene silencing is now widely used in biomedical research and clinical applications, efficient intercellular delivery of antisense RNA remains a challenge. Here, we describe an arrestin domain–containing 1–mediated microvesicle-based platform for the silencing of gene expression.

## Introduction

Precise and reversible suppression of gene expression by antisense RNA sequences is a strategy that is widely used in both basic research and clinical medicine (for reviews, see 1–3). Naked or unmodified antisense RNA is rapidly degraded in biological fluids and is inefficiently internalized by cells (4, 5). Consequently, antisense RNAs used to silence gene expression have been delivered by encapsulating them extracellularly in lipid-based or gold-based nanoparticles (6, 7) or in vesicles released from cells grown in culture (8–11). However, consistent and quantitatively reproducible RNA encapsulation in particles formed extracellularly has been a challenge (12, 13).

Arrestin domain–containing 1 (ARRDC1)-mediated microvesicles (ARMMs) are a naturally occurring distinct subclass of extracellular vesicles (EVs) that are uniquely dependent on the production of the arrestin-like cellular protein ARRDC1, and which—unlike other vesicles released from cells—are formed by outward budding of cytoplasmic membranes (14). During ARMMs formation, the ARRDC1 protein is attached to ARMMs membranes and is loaded into ARMMs—carrying along other macromolecules linked to it (14, 15). ARMMs can fuse with the plasma membranes of cells they encounter, and ARMMs cargos are discharged directly into the cytosol of recipient cells instead of being internalized by endocytosis—thus avoiding degradative actions of endosomal enzymes (14, 15). Collectively, these properties have prompted investigation of ARMMs' abilities to deliver biologically manufactured macromolecules intercellularly (15–17).

Here, we describe and test a platform that synthesizes short hairpin RNA (shRNA) prodrugs as biochemically inert modules that are protected from RNA processing and degradation during their production and loading into ARMMs, but which become active gene-silencing agents upon delivery to recipient cells.

## Results

### Selective loading of shRNA into ARMMs

**Figure 1:**
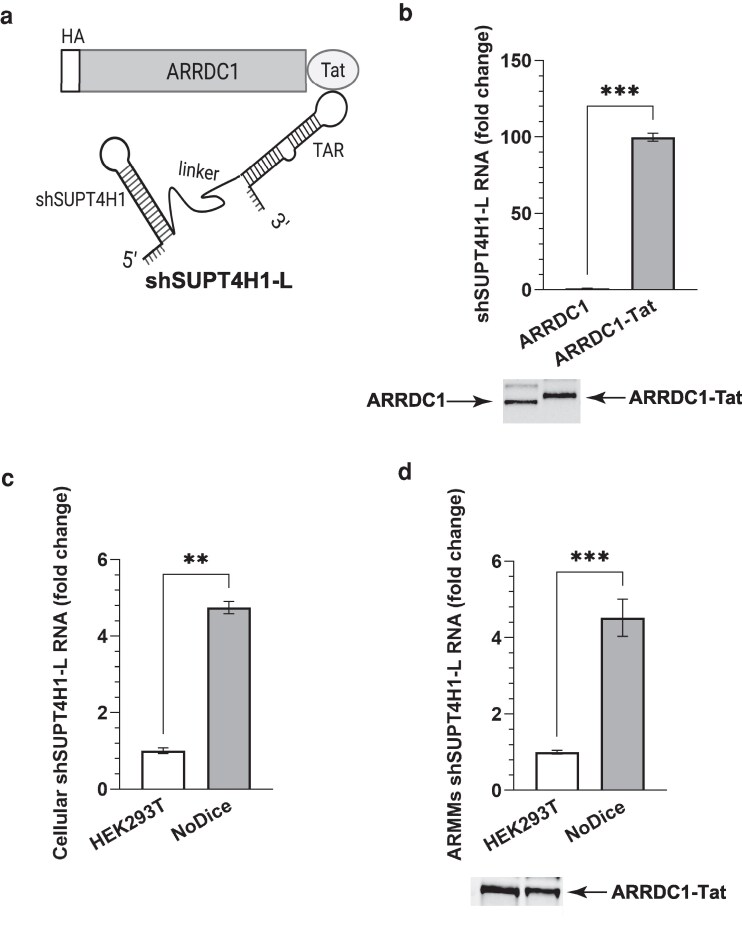
Effect of TAR–Tat interaction and DICER1 mutation on shRNA loading into ARMMs. a) Schematic representation of strategy used for packaging shSUPT4H1-L RNA into ARMMs. pSuper-shSUPT4H1-L, which expresses the transcript containing an shRNA targeting SUPT4H1 messenger RNA, a 46-nucleotide linker, and a 68-nt TAR element, was co-transfected with a construct expressing an HA-tagged ARRDC1–Tat (pcDNA3–HA-ARRDC1–Tat) fusion protein into the HEK293T cells. Interaction between TAR and the Tat peptide is shown in the diagram. b) Effect of the TAR–Tat interaction on loading of shSUPT4H1-L. Fold enrichment of shSUPT4H1-L RNA in ARMMs from HEK293T cells co-transfected with shSUPT4H1-L and pcDNA3–HA-ARRDC1–Tat is shown relative to ARMMs from cells co-transfected with shSUPT4H1-L and pcDNA3–HA-ARRDC1. Quantification was done by qRT-PCR, and values were normalized according to approximate concentrations of ARMMs, which were quantified by Western blotting analysis of ARRDC1 or ARRDC1–Tat abundance on ARMMs preparations (lower panel). c and d) Comparison of the fold change of shSUPT4H1-L RNA produced by HEK293T and NoDice detected in cells (c) and in ARMMs (d). HEK293T and NoDice cells were transiently co-transfected with pSuper-shSUPT4H1-L and pcDNA3–HA-ARRDC1–Tat. RNAs were purified from transfected cells at the time when ARMMs were collected. GAPDH mRNA was used for normalization of cellular RNA levels for qRT-PCR quantification. The bar graphs indicate the fold change of the shSUPT4H1-L RNA from NoDice compared with HEK293T. The lower panel in (d) shows the Western blot of ARRDC1–Tat protein to monitor the amount of collected ARMM particles from each preparation and serves as an internal control for RNA input normalization. b–d) Data were collected from three biological replicates: **P < 0.01, ***P < 0.001.

During the formation of ARMMs by budding of the plasma membrane, portions of the cell cytoplasm and cytoplasmic RNAs are transferred nonspecifically to ARMMs (14). Additionally, mRNAs can be loaded specifically into ARMMs by linking them to the trans-activation response (TAR) element of HIV-1, and concurrently expressing a fusion protein consisting of the TAR-binding HIV-1 trans-activator of transcription (Tat) peptide and the cellular protein ARRDC1 (15). We wished to produce ARMMs that package and deliver biologically manufactured antisense transcripts and investigated the applicability of Tat–TAR linkage to the packaging of highly structured shRNA that targets mRNA encoding the transcription elongation protein SUPT4H1 (shSUPT4H1-L, Fig. 1a). As shown in Fig. 1b, loading of the TAR-linked shRNA, shSUPT4H1-L, was increased more than 100-fold by co-expression of a fusion protein containing ARRDC1 and Tat components. In this and all subsequent experiments, ARMMs abundance was normalized according to ARRDC1 levels detected by Western blotting. We chose ARRDC1 as a marker for quantification because it is a defining structural feature of ARMMs, whereas total EV particle counts do not distinguish ARMMs from other EVs.

As processing and degradation of shRNA upon binding to complementary RNA normally are initiated by endoribonucleolytic cleavage by the RNase III family member DICER1 (18), we tested the effects of a DICER1 mutation (NoDice cells) (19) on shSUPT4H1-L RNA. As seen in Fig. 1c and d, mutation of DICER1 resulted in a multifold increase in the concentration of shSUPT4H1-L RNA in both the cells that synthesize the shRNA and in ARMMs released from those cells (Fig. 1d). NoDice cells were therefore used for the production of ARMMs and shRNAs in our subsequent studies.

### Embedding TAR within the shRNA loop enables loading and functional delivery of shRNA molecules by ARMMs

**Figure 2:**
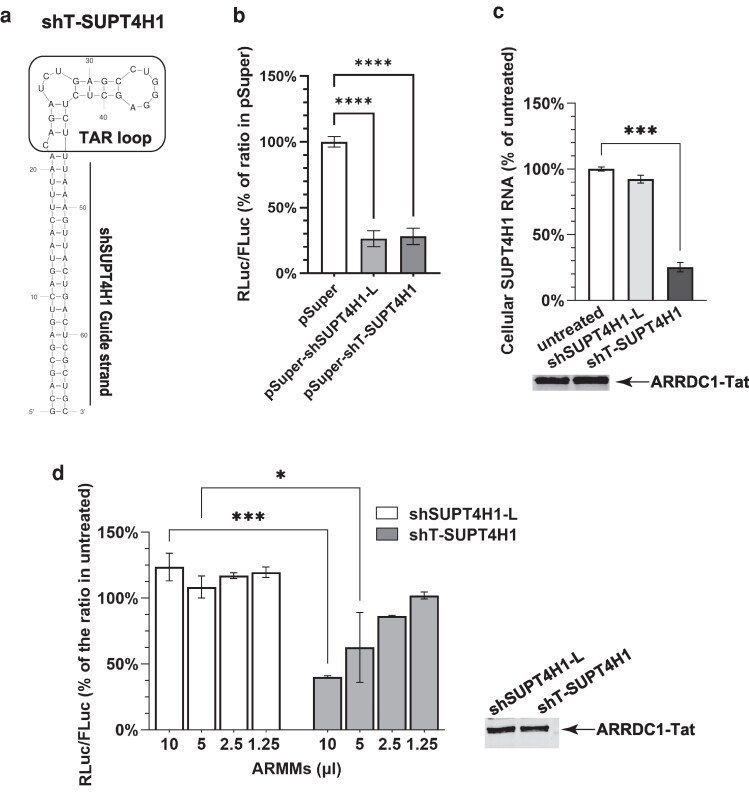
Effects of embedding TAR sequence within the shRNA framework. a) Sequence and predicted secondary structure of shT-SUPT4H1 RNA. The 24-nucleotide TAR connecting the two limbs of the shRNA stem region is shown. b) Knockdown of shSUPT4H1-L and shT-SUPT4H1 constructs was evaluated using a dual-luciferase reporter assay as described in Materials and methods. The reporter plasmid psiCHECK-2 contains the entire SUPT4H1 coding region inserted at the 3′ end of the RLuc gene. FLuc served as a transfection control. Normalized luciferase activity is presented as the RLuc/FLuc ratio and was compared with values obtained from cells transfected with the pSuper plasmid, which served as the no-knockdown control. c) Knockdown of endogenous SUPT4H1 in U2OS cells following delivery of ARMMs containing shT-SUPT4H1 or shSUPT4H1-L. Relative SUPT4H1 mRNA level in U2OS cells exposed to approximately equal amounts of ARMMs that have been manufactured by NoDice cells expressing shSUPT4H1-L or shT-SUPT4H1 RNA compared with untreated U2OS cells. Treated cells were exposed to equal volumes of ARMMs and cultured for 40 h before RNA extraction. SUPT4H1 mRNA was quantified by qRT-PCR, using GAPDH mRNA for normalization of cellular mRNA measurements. The lower panel shows Western blotting data for the ARRDC1–Tat protein, which was used to confirm that similar numbers of ARMMs particles were present in the samples. Bar graphs show the average of results collected from three separate experiments. d) Knockdown of SUPT4H1 by ARMMs containing shT-SUPT4H1 or shSUPT4H1-L in U2OS cells using the same dual-luciferase reporter assay as (b). RLuc/FLuc ratios were compared with values obtained from cells that did not receive ARMMs treatment. b–d) Data were collected from three biological replicates: *P < 0.05, ***P < 0.001, ****P < 0.0001.

In the experiments described above, shRNA was loaded into ARMMs using a full-length TAR element positioned at the 3′ terminus of the shRNA and separated from it by a defined linker sequence, as has been done to load mRNA into ARMMs (15). However, this strategy allows for ribonucleolytic cleavages occurring within the intervening linker region to disrupt physical continuity between the shRNA and TAR segments and consequently could limit RNA loading into ARMMs. We strategized that embedding a TAR sequence within the structural framework of shRNAs would address that concern and, importantly, could also provide modularity to the loading of shRNAs. We chose to use an embedded 24-nucleotide segment of TAR that was shown by Unwalla and Rossi (20) to be sufficient for interaction between shRNA and Tat, as an shRNA loop region, and designated transcripts containing such embedded TAR sequences as shT-RNAs. As shown in Fig. 2a, transcript length was reduced from >160 nucleotides in shSUPT4H1-L to <70 nucleotides in shT-SUPT4H1 RNA.

Earlier studies have shown that shRNA sequence and secondary structure can affect shRNA processing and other RNA–protein interactions (21–24). However, we found that shSUPT4H1-L and shT-SUPT4H1, which target the same SUPT4H1 RNA sequence, had similar gene-silencing capabilities, as determined by psiCHECK-2 dual reporter system (Promega) analysis, when introduced into U2OS cells directly by transient transfection (Fig. 2b). Notwithstanding inherently similar gene-silencing abilities of these shRNA constructs, ARMMs derived from cells expressing them had strikingly different abilities to silence gene expression: shT-SUPT4H1 reduced the expression of SUPT4H1 in recipient U2OS cells, whereas ARMMs derived from cells expressing shSUPT4H1-L RNA did not produce detectable knockdown (Fig. 2c and d). Together, these findings support the notion that eliminating the previously used linker segment and embedding TAR within the shRNA framework enhances Tat–TAR–mediated loading of shRNA into ARMMs.

To enhance ARMMs budding and uptake, the ARMMs used in these and subsequent experiments were pseudotyped with vesicular stomatitis virus glycoprotein (VSV-G), which has previously been shown to enhance ARMMs budding and augment ARMMs-mediated delivery (17).

### Delivery of antiviral shT-RNA prodrug modules by ARMMs

To elucidate more fully the effects of the above manipulations and to specifically learn whether the platform we designed for delivery of shT-RNAs by ARMMs can deliver antisense RNA in sufficient amounts to exhibit antiviral activity, we chose SARS-CoV-2 virus RNA, which has been studied extensively as a potential target for antisense RNAs (25, 26), as the shT-RNA target. Earlier studies have identified two highly conserved regions of the SARS-CoV-2 virus genome that are essential for virus replication (27–29): the untranslated region (UTR) located near the 5′ end of the genome (30) and the region encoding the virus's RNA-dependent RNA polymerase (RDRP) (31, 32). We sought to determine whether shT-RNAs directed against these regions can, like shT-SUPT4H1, be loaded into ARMMs and can subsequently be processed into biologically active antiviral RNA.

Using previously published data (30) and the Integrated DNA Technologies (IDT) small interfering RNA (siRNA) design tool to analyze SARS-CoV-2 genomic RNA, we generated 22 distinct shT-RNAs whose loop segments contain the 24-nucleotide TAR-derived sequence and whose stem regions corresponded to sequences within either the 5′UTR or RDRP of SARS-CoV-2. The complete list of shRNA target sequences is shown in Table S1. Screening revealed a range of knockdown efficiencies (Fig. S1). Based on the extent of target suppression by these sequences, one shRNA targeting the 5′UTR (shT-5′UTR2) and one targeting RDRP (shT-RDRP8) were selected for further study. We found that ARMMs loaded with each of these shT-RNAs inhibited SARS-CoV-2 replication in virus-infected cells (Fig. S2).

**Figure 3:**
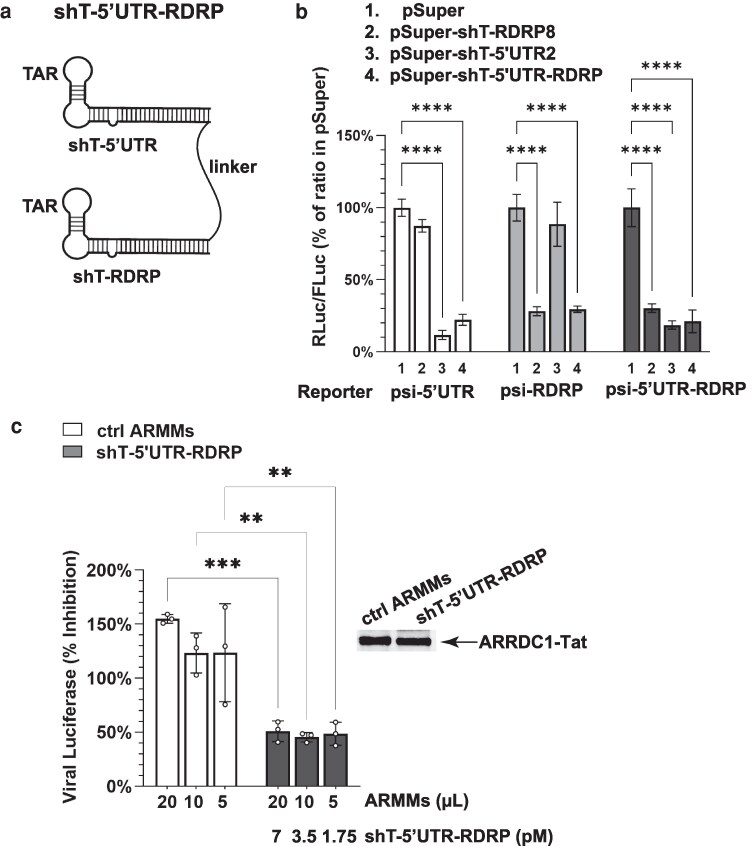
Effects of shT-RNA constructs on SARS-CoV-2 gene expression and on virus replication. a) Schematic representation of the tandem two-component shT-5′UTR-RDRP construct, which targets the 5′UTR and RDRP regions of the SARS-CoV-2 genome. The two shT-RNA modules are linked in tandem by a 34-nucleotide spacer with a randomly determined sequence predicted by the RNAfold program to have minimal secondary structure. Transcripts were synthesized from plasmid templates introduced into HEK293T NoDice cells. b) Evaluation of the relative knockdown of component modules of shT-5′UTR-RDRP as determined by the dual-luciferase reporter assay. Plasmids expressing shT-5′UTR2, shT-RDRP8, or the tandem shT-5′UTR-RDRP construct were individually co-transfected with psiCHECK-2 reporter plasmids containing SARS-CoV-2 5′UTR, RDRP, or combined target sequences cloned 3′ to the RLuc. FLuc served as an internal control for normalization of transfection efficiency. Gene silencing was quantified as the *Renilla*/Firefly (RLuc/FLuc) ratio and expressed relative to cells transfected with the pSuper control plasmid. c) Inhibition of SARS-CoV-2 replication by ARMMs-delivered shT-5′UTR-RDRP RNA. The concentrations of shT-5′UTR-RDRP RNA in the ARMMs preparations used for treatment were as indicated. These values were quantified by qRT-PCR using a standard curve generated from known amounts of in vitro–synthesized shT-5′UTR-RDRP RNA (see Materials and methods and Fig. S3 for details). The primer pair used for quantification spans both shT-RNA modules and the linker, thereby ensuring detection of the intact tandem transcript. ARMMs preparations from cells transfected with pSuper served as negative controls and were normalized to ARRDC1–Tat levels determined by Western blotting (right panel). Data represent three biological replicates in (b) and (c). Statistical significance was determined by one-way ANOVA followed by Dunnett's multiple comparisons test; **P < 0.01, ***P < 0.001, ****P < 0.0001.

We engineered a transcript in which the two shT-RNA modules we designed were linked in tandem (shT-5′UTR-RDRP; Fig. 3a). Using the psiCHECK-2 dual-luciferase reporter assay, as described in Materials and methods, we assessed the gene-silencing activity of the components of this multicomponent transcript and found that knockdown by the two modules was not additive (Fig. 3b). Importantly, each targeting module within the tandem construct retained the ability to suppress its respective target sequence (Fig. 3b), indicating that incorporation into a single transcript does not compromise individual shT-RNA function. Moreover, we found that ARMMs carrying this multicomponent antiviral construct effectively inhibited SARS-CoV-2 replication (Fig. 3c). The ability to concurrently target separate regions of the virus genome using modular shT-RNA elements synthesized as components of a single transcript may offer a potential approach to mitigate the development of virus resistance.

### Creating a scalable clonal cell factory that manufactures ARMMs containing shT-RNA prodrugs

The SARS-CoV-2 experiments described above rely on transient transfection of plasmid DNA to overexpress ARRDC1–Tat and TAR-embedded shT-RNAs. To establish a stable cell line capable of producing ARMMs loaded with shT-RNA, clones of NoDice HEK293T cells that we had engineered to express high levels of ARRDC1–Tat fusion protein were screened by qRT-PCR to identify isolates that express high levels of shT-RNA modules encoded by plasmids introduced by transfection. A cell clone designated as WYS222 was selected for propagation and further characterization.

**Figure 4:**
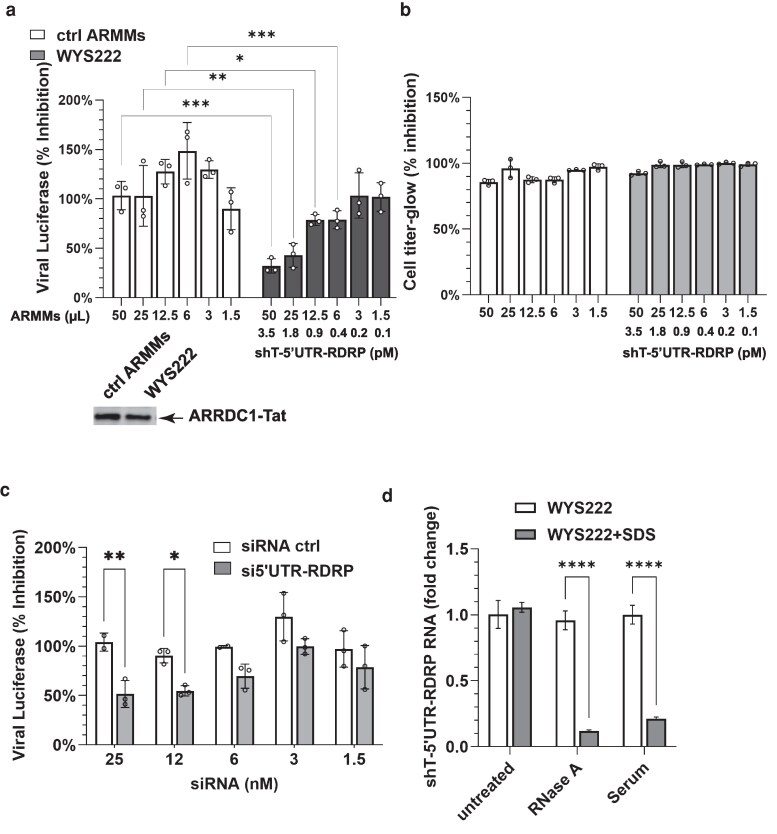
Effects of ARMMs produced by a clonal cell line WYS222. a) Viral replication assays in cells treated with ARMMs. The lower panel shows the Western blot detection of the ARRDC1–Tat protein used for normalization. The volume of ARMMs ranged from 50 to 1.5 μL in 1:1 serial dilution. The respective concentration of shT-5′UTR-RDRP ranged from 3.5 to 0.11 pM. b) Cell viability after virus infection is presented as a percentage compared with viability data from untreated virus-infected cells. c) Viral replication in cells treated by siRNAs. siRNAs were transfected immediately before the virus infection. The scrambled siRNA sequence that served as the negative control was designed by and ordered from IDT. d) Effect of SDS (gray bars) on the ability of ARMMs to protect shT-5′UTR-RDRP from degradation. One percent SDS was used to disrupt ARMMs membranes before incubation with RNase A or serum. Relative amounts of shT-5′UTR-RDRP RNA were determined by qRT-PCR as described in Materials and methods. Bar graphs show the percentage of RNA in SDS-treated vs. untreated (white bars) ARMMs. a–d) Data were collected from three biological replicates: *P < 0.05, **P < 0.01, ***P < 0.001, ****P < 0.0001.

To facilitate production of ARMMs from the WYS222 clonal cell line, we wanted to establish culture conditions that allow these HEK293T-derived cells to be propagated transiently in suspension following transfer from conventional surface-attached cultures (see Materials and methods). Initial attempts to generate permanently suspension-adapted derivatives of the WYS222 line resulted in reduced cell viability and ARMMs production during extended suspension culture. We therefore developed a transient suspension production procedure in which cells recovered from culture plates could be transferred directly into suspension cultures in shaking, baffled flasks. Under the conditions we used, cells continued to produce ARMMs efficiently in liquid cultures for at least 72 h, enabling routine recovery of ARMMs-containing culture supernatants from volumes of several hundred milliliters without the need to generate stable suspension-adapted producer lines. The resulting ARMMs preparations were used for the experiments shown in Fig. 4a and B. As did ARMMs produced by transfected cells (Fig. 3c), ARMMs produced by WYS222 inhibited SARS-CoV-2 replication (Fig. 4a), and addition of these vesicles to infected cells did not exhibit detectable cytotoxicity (Fig. 4b).

To learn the relative antisense activity of WYS222 vs. antisense activity of the same sequence of nucleotides carried by chemically synthesized siRNA introduced by transfection using lipid-based nanoparticles, we determined the effects of these agents on SARS-CoV-2 virus propagation. Although differences in the methods of delivery precluded direct comparisons of molar efficacy, we observed that achieving a comparable antiviral effect required a >1,000-fold higher concentration of chemically synthesized siRNA (pM of shT-RNA vs. nM of siRNA, Fig. 4a and c).

Previous studies have shown that the lipid-containing membrane-encapsulating EVs shields chemically synthesized RNA cargos from ribonuclease-mediated degradation (33, 34). The data shown in Fig. 4d indicate that shT-RNA synthesized by a cellular factory and loaded into nascent ARMMs is similarly shielded by ARMMs membranes after release of ARMMs into the extracellular environment. Disruption of the ARMMs membrane by treatment with sodium dodecyl sulfate (SDS), a detergent that dissolves lipid membranes, eliminated protection.

## Discussion

We have reported the development and testing of an ARMMs-based platform that can protect an inert antisense RNA prodrug from ribonucleolytic attack during its intracellular synthesis and loading into nascent EVs while enabling conversion of the prodrug into an active gene-silencing agent when delivered to its cellular target. The procedures required to produce and deliver such prodrugs are simple and robust: (i) embedding within the RNA framework a sequence that enables loading of shT-RNA into ARMMs being manufactured by the same cell, (ii) synthesizing both ARMMs and ARMMs cargos in cells that lack the RNase III family endoribonuclease DICER1, and (iii) concurrently overproducing an ARRDC1 protein fused with a peptide that interacts with the embedded sequence. Synthesizing shRNA in DICER1 mutant cells prevents its rapid conversion into an siRNA that could attack cognate mRNAs made by the same cell and also its possible destruction by encountering an off-target RNA. Integrating TAR element sequences within the structural framework of individual shRNAs provides the opportunity for modular loading in addition to ensuring continuity of the antisense segment with the element that enables its loading into ARMMs.

The ability to incorporate multiple RNA prodrugs as distinct modules may facilitate simultaneous targeting of disparate genes and, in antiviral applications, may mitigate the emergence of resistance or enable coordinated inhibition of co-infecting pathogens. Our results raise the prospect that additional manipulations of RNA-processing enzymes may further enhance the protection and stability of prodrug RNAs.

As chemically synthesized antisense nucleic acids delivered by synthetic nanoparticles are internalized by endocytosis and then are transferred to lysosomes, they are subjected to degradation by enzymes of these internal organelles (35, 36). Chemical modifications have been used to protect nucleic acids from nucleolytic enzymes while they are being delivered to the cytosol of recipient cells (37, 38). In contrast, RNAs delivered by ARMMs enter the cytoplasm directly through fusion of ARMMs membranes with the plasma membranes of cells they interact with (14, 15), bypassing lysosomal degradation and the need for protective chemical modification of the nucleic acid. As we have shown, the membrane that encapsulates ARMMs that have been released from cells can protect their prodrug RNA cargo from degradation by ribonucleases in the surrounding environment.

VSV-G previously has been found to enhance ARMMs budding and to promote fusion of ARMMs membranes with recipient cell plasma membranes (17). VSV-G was therefore co-expressed in ARMMs-producing cells in all of our experiments.

We have shown that the platform we have designed is applicable to delivery of antisense prodrugs by ARMMs produced by clonal cell lines as well as by ARMMs produced by cells transiently transfected by plasmids that manufacture both ARMMs and ARMMs cargos. Transient transfection typically results in high gene copy numbers and elevated short-term expression of gene products, whereas stable clonal cell lines generally harbor a limited number of integrated transgene copies. In cell populations induced to generate ARMMs and shRNA by transient transfection, the copy number of transgenes inevitably varies from cell to cell and from experiment to experiment, whereas in a clonal cell line, transgene copy number is uniform. Thus, data obtained for clonal cell lines vs. transiently transfected cells cannot be directly compared.

Collectively, the findings reported here suggest that ARMMs-mediated delivery of antisense RNA in a prodrug form offers a strategy for the silencing of gene expression without affecting genome integrity.

## Materials and methods

### Cell culture and transfection

Human HEK293T, NoDice, A549-hACE2, and U2OS cells were routinely maintained in Dulbecco's Modified Eagle Medium (DMEM) (Corning Inc.) supplemented with 10% fetal bovine serum (FBS; Omega Scientific, Inc.). NoDice cells expressing ARRDC1–Tat protein were cultured in a media containing 50% ESF SFM^TM^ mammalian cell culture medium (Expression Systems LLC), 45% DMEM, and 5% FBS plus 10 μg/mL blasticidin S hydrochloride (BSD). The clonal cell line for WYS222 production was maintained in 50% ESF SFM^TM^ mammalian cell culture medium, 45% DMEM, and 5% FBS plus 10 μg/mL BSD and 2 μg/mL puromycin in a plate and in 75% ESF SFM^TM^ mammalian cell culture medium, 22.5% DMEM, and 2.5% FBS in suspension culture. In experiments where ARMMs were collected, exosome-depleted FBS was made by ultracentrifugation at 32,000 rpm for 2 h and then filtered through a 0.2 μm filter. Lipofectamine 3000 (Thermo Fisher Scientific) was used for plasmid transfection, and Lipofectamine RNAiMAX (Thermo Fisher Scientific) was used for siRNA transfection.

### Plasmids

pcDNA3–ARRDC1 and pcDNA3–ARRDC1–Tat were described previously (15). The human influenza hemagglutinin (HA)-tag at the N-terminus of ARRDC1 enables the detection of ARRDC1 using anti-HA antibody. To add the Puro marker to the pSuper plasmid (39), the PGK promoter and Puro gene were amplified by PCR from the pLKO.1 puro plasmid template and inserted into pSuper plasmid position 2189 with NEBuilder HiFi DNA Assembly Cloning Kit (New England Biolabs). shRNA fragments were chemically synthesized and inserted into pSuper-Puro between the unique BglII and HindIII sites 3′ to the H1 promoter. The entire RDRP gene fragment was purchased from IDT and cloned into pcDNA3.1 (+). For psiCHECK-2 reporter assay, gene fragments were generated by PCR and cloned 3′ to the stop codon of *Renilla* luciferase (RLuc) at the XhoI and NotI restriction enzyme sites of the psiCHECK-2 plasmid.

Primers used for PCR are listed in Table S1.

### ARMMs production by transient transfection

HEK293T and NoDice cells (5 × 10^6^) were seeded onto a 10-cm plate 24 h prior to transfection by a total of 5 μg plasmid DNA that included 1.5 μg of pcDNA3–ARRDC1–Tat, 3 μg of pSuper-shRNA, 0.4 μg of VSV-G (to enhance ARMMs production and absorption (17, 40, 41)), and 0.1 μg of pEGFP-C1 (to monitor transfection efficiency). Sixteen hours posttransfection, the transfected cells were trypsinized and transferred to a 15-cm plate containing 50% ESF SFM^TM^ mammalian cell culture medium, 45% DMEM, and 5% exosome-depleted FBS. After an additional 48 h of culture, the media were collected for downstream ARMMs purification.

### ARMMs purification and quantification

ARMMs were purified by differential centrifugation, as described previously (15, 42). The supernatant collected from cultured cells was centrifuged at 6,000 rpm for 15 min, then filtered through 0.22 μm cellulose acetate filters, and subjected to centrifugation, using a cushion of 4 mL 30% sucrose at the bottom of the ultracentrifuge tube, at 32,000 rpm in an SW 32Ti rotor (Beckman Coulter) for 2 h. The supernatant above and the interphase of the 30% sucrose cushion were aspirated, and the remaining sucrose solution was diluted with PBS before being centrifuged again at 32,000 rpm in the SW 32Ti rotor for 2 h. After this step, the supernatant was aspirated, and the pellet was resuspended in DMEM and stored at 4 °C.

For RNase and serum treatment, the purified ARMMs were incubated at 37 °C for 15 min with 10 pg/μL of DNase-free RNase A (Thermo Fisher Scientific) or 20% v/v serum. One percent SDS was used to destroy the ARMMs membrane and expose the cargo RNAs for environmental degradation.

Quantification of ARMMs was based on ARRDC1 concentration in samples. ARRDC1 abundance was determined by Western blotting analysis. Three microliters of purified ARMMs were mixed with lysis buffer (30 mM Tris-HCl, pH 8.0, 5 mM NaCl, 0.1% Triton X-100), 4× XT sample buffer, and 20× XT Reducing Agent (Bio-Rad) and incubated at 70 °C for 10 min before running on 4–12% Criterion XT precast gel (Bio-Rad). Protein in the gel was transferred to a polyvinylidene fluoride (PVDF) membrane, and Western blotting was performed with rabbit mAb anti-HA antibody directed against ARRDC1 (C29F4, Cell Signaling Technology).

### RNA extraction and quantification

Vazyme MiPure cell/tissue miRNA Kit was used to purify RNA from ARMMs. Briefly, 250 μL of RNA isolator was added to 20–50 μL of ARMMs before adding 100 ng of tomato RNA, which served as experimental spike-in control. After the RNA purification procedure according to the manufacturer's protocol, 30 μL nuclease-free H_2_O was used for elution from columns, and 6 μL eluted RNA was used for reverse transcription (RT) with HiScript III RT SuperMix for qPCR (Vazyme).

For the quantification of the absolute amount of shRNA packaged in ARMMs, various quantities of T7-transcribed shRNA were used to generate a standard curve. The corresponding shRNA sequence was amplified by PCR using primers containing T7 promoter sequences. The amplified DNA fragment was gel purified and extracted with the QIAquick Gel Extraction Kit (Qiagen) and transcribed with the MAXIscript T3/T7 Transcription Kit (Invitrogen). The reaction product was purified with the MiPure cell/tissue miRNA kit and then subjected to NanoDrop (Thermo Fisher Scientific) for RNA quantification. Serial dilutions (1:3) of 500 pg of in vitro–synthesized RNA supplemented by 100 ng of tomato RNA were subjected to the procedures used for ARMMs RNA purification and RT-PCR.

For real-time PCR, 1/20 of reverse-transcribed cDNA product was used with Taq Pro Multiple Probe qPCR Mix (Vazyme). The assay was run and detected with QuantStudio 3 (Fisher Scientific).

### psiCHECK2 dual-luciferase reporter assay

Cells were seeded in 96-well plates at a density between 1.5 and 2.5 × 10^4^ cells/well, depending on cell type, 24 h before transfection. To measure knockdown capability of ARMMs, various amounts of ARMMs and culture media to a total volume of 50 μL were added to the cells. siRNAs used as positive controls were introduced by transfection using Lipofectamine RNAiMAX (Thermo Fisher Scientific). After 1 h, 20 ng of the reporter plasmid was introduced by Lipofectamine 3000 (Thermo Fisher Scientific)-mediated transfection according to the manufacturer's instructions. To assess knockdown capabilities by transfected plasmids, reporter plasmid DNA and shRNA plasmid DNA were mixed in a 1:25 ratio and 50 ng of the plasmid mix was co-transfected using Lipofectamine 3000. Sixteen hours after transfection of the reporter plasmid firefly luciferase (FLuc) and RLuc activities were measured sequentially using Dual-Glo Luciferase Assay System (Promega) and plate reader Infinity M200 (Tecan). Relative light units (RLUs) generated by RLuc were normalized according to RLU generated by the FLuc which serves as a control for variation in transfection efficiency. Knockdown was determined by comparison of the RLuc/FLuc ratio in treated cells with the RLuc/FLuc ratio observed for cells transfected with reporter plasmid only. The reported results are shown in figures obtained by three or more experiments.

### SARS-CoV-2 virus nano-luciferase assay

All SARS-CoV-2-related infection experiments were performed under Biosafety Level 3 (BSL-3) conditions using enhanced respiratory personal protection equipment at Stanford University and Vitalant Research Institute (San Francisco). Freshly prepared A549-hACE2 cells were seeded on 96-well plates at a density of 1 × 10^4^/well and grown for 24 h. Aliquots of ARMMs or siRNA transfection mixture were added to cultures of cells that were subsequently infected with SARS-CoV-2-Nluc virus (43) at multiplicity of infection (MOI) of 0.01, and luciferase activity generated by virus propagation was assayed 24 h after virus infection. Data obtained for untreated virus-infected cells were used as a control to assess the effects of virus replication on the assay. For every nano-luciferase assay plate, a duplicate plate treated in the same way was used to measure cell viability as determined by the CellTiter-Glo luminescent cell viability assay (Promega). Viability data were used for normalization of data according to the number of viable cells.

### Selection of ARMMs-producing cell clones and suspension culture conditions for ARMMs collection from clonal cells

NoDice cells were transfected with pcDNA3–ARRDC1–Tat plasmid, and 10 μg/mL BSD was used for the selection of ARRDC1–Tat expression clones. Western blot analyses were used to screen for the cell clone with the highest expression level of ARRDC1–Tat. The selected NoDice–ARRDC1–Tat clonal cells were then transfected with pSuper-shT-5′UTR-RDRP plasmid and selected with 2 μg/mL puromycin. Individual colonies were picked for expansion, and their cellular expression of shT-5′UTR-RDRP RNA was determined by real-time qRT-PCR. Several high-expression clones were selected for further evaluation. ARMMs were purified from these clones, which were grown at the same cell seeding number, and their shT-RNA cargos were quantified. The clonal cell line with the highest amount of shT-RNA cargo was chosen and designated as WYS222.

Clonal cells (5 × 10^6^) were seeded onto 10 cm plates 24 h prior to transfection with 1 μg of plasmid DNA expressing VSV-G. Sixteen hours posttransfection, the cells were trypsinized and pooled into a sterile baffled Erlenmeyer flask with 30 mL of culture media (75% ESF SFM^TM^ mammalian cell culture medium, 22.5% DMEM, and 2.5% FBS) for each 10 cm plate to create a shaking suspension culture. After 48 to 72 h of cell growth, the culture media were collected for ARMMs purification.

### Statistical analysis

All experiments included biological replicates, and the sample sizes for each experimental group under various conditions are detailed in the corresponding figure legends and Materials and methods section. To evaluate statistical significance, t tests were employed for comparisons between two groups, while one-way ANOVA was utilized for comparisons involving more than two groups. A *P*-value of <0.05 was deemed statistically significant, with *P*-values represented as follows: *P < 0.05, **P < 0.01, ***P < 0.001, ****P < 0.0001. The data are expressed as means ± SEM. All statistical analyses were conducted using GraphPad Prism 8.0.

## Supplementary Material

pgag121_Supplementary_Data

## Data Availability

All data referred to in this report are included in the manuscript and Supplementary material.
