# Supplementary material for: Cell factories that manufacture microvesicles containing gene silencing RNA prodrugs
Source: PNAS Nexus. 2026 Apr 17;5(5):pgag121. doi: 10.1093/pnasnexus/pgag121 (PMC13148645; doi:10.1093/pnasnexus/pgag121)
Supplement: pgag121_Supplementary_Data [file pgag121_supplementary_data.pdf]

<u>**Supplementary Material**</u>

Cell Factories That Manufacture Microvesicles Containing Gene Silencing RNA Prodrugs

Yanan Feng[a,1], Weijing Xu[a,1], Ning Deng[a,b], Jing Jin[c], Xiaohong Tang[a], Jaishree Garhyan[d], Stanley N. Cohen[a,e,*]

[a]Department of Genetics, Stanford University School of Medicine, Stanford, CA, USA.

[b]Current Address: BridGene Biosciences, Inc., San Jose, CA, USA.

[c]Vitalant Research Institute, San Francisco, CA, USA.

[d]In Vitro Biosafety Level 3 (BSL3) Service Center, Stanford University School of Medicine, Stanford, CA, USA.

[e]Department of Medicine, Stanford University School of Medicine, Stanford, CA, USA.

*To whom correspondence should be addressed: Email: sncohen@stanford.edu

[1]Y.F. and W.X. contributed equally to this work and have been listed alphabetically.

**CONTENTS**	**PAGE**
**Fig. S1.** Screening for shT-RNA sequences targeting the RDRP gene and 5′UTR of SARS-CoV-2 with the psiCHEK-2 dual-luciferase assay	**2**
**Fig. S2.** Inhibition of SARS-CoV-2 replication by ARMMs carrying shT-RNAs targeting either RDRP or 5′UTR	**3**
**Fig. S3.** Standard curve for quantification of shT-5′UTR-RDRP RNA by qRT-PCR	**4**
**Table S1.** shRNA Targets and Quantitative PCR Primers	**5**

Figure S1

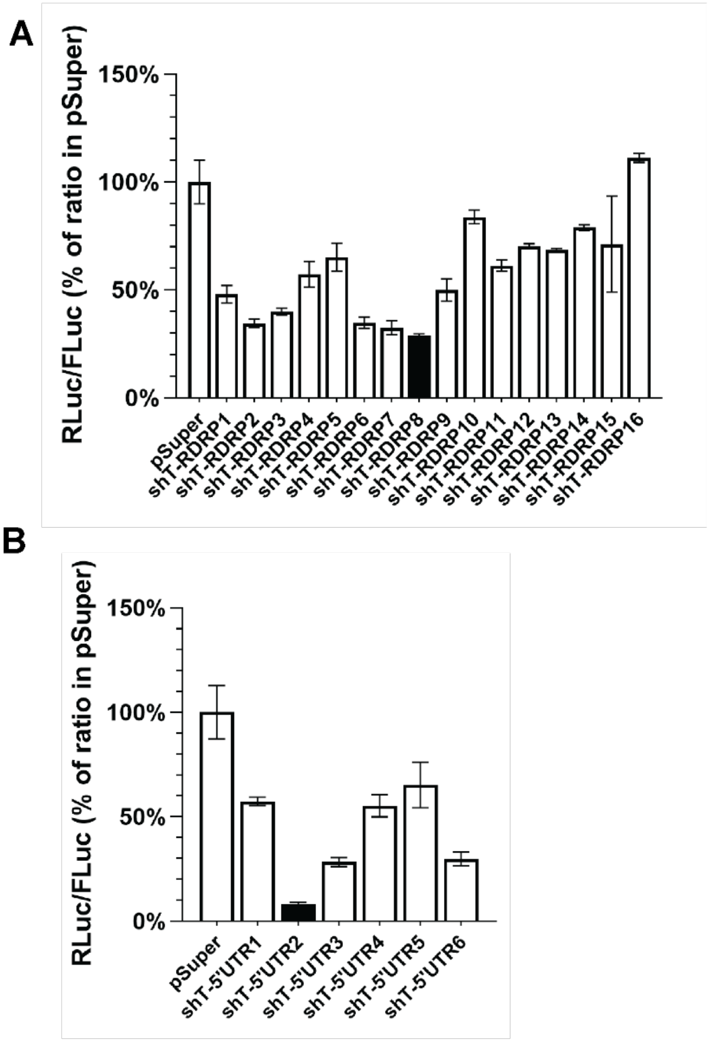

Fig. S1. Screening for shT-RNA sequences targeting the RDRP gene and 5′UTR of SARS-CoV-2 with the psiCHEK-2 dual-luciferase assay. (A) Screening of shT-RDRP constructs**.** (B) Screening of shT-5′UTR constructs. HEK293T cells were co-transfected with individual shT-RDRP or 5′UTR constructs and a psiCHECK-2 reporter plasmid containing either the full-length SARS-CoV-2 RDRP coding sequence or 5′UTR cloned downstream of the *Renilla* luciferase gene. Firefly luciferase expressed from the same plasmid served as an internal normalization control. Gene-silencing efficiency was determined by calculating the *Renilla*/Firefly (RLuc/FLuc) ratio and normalizing values to cells transfected with the empty pSuper vector (set to 100%). Data represent the mean ± SD from three biological replicates. Statistical analysis was performed using one-way ANOVA followed by Dunnett's multiple comparisons test. All shT-RNA constructs that reduced reporter activity by $> 30\%$ showed p value < 0.0001.

Figure S2

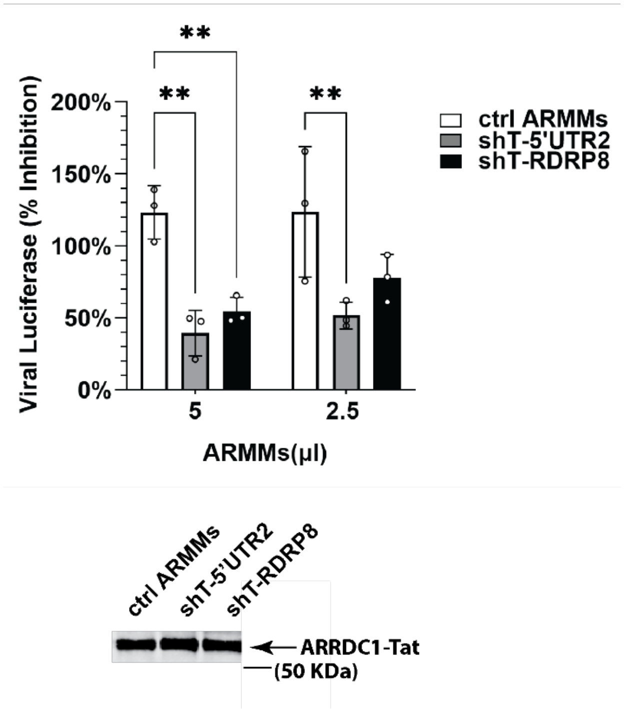

Fig. S2. Inhibition of SARS-CoV-2 replication by ARMMs carrying shT-RNAs targeting either RDRP or 5′UTR. The graph shows virus luciferase ratio determined by comparing results obtained following ARMMs treatment with no ARMMs/virus only controls. Lower panel shows the Western blot detection of ARRDC1-Tat protein used for normalization. Statistical significance was determined by one-way ANOVA followed by Dunnett’s multiple comparisons test; **p < 0.01.

Figure S3

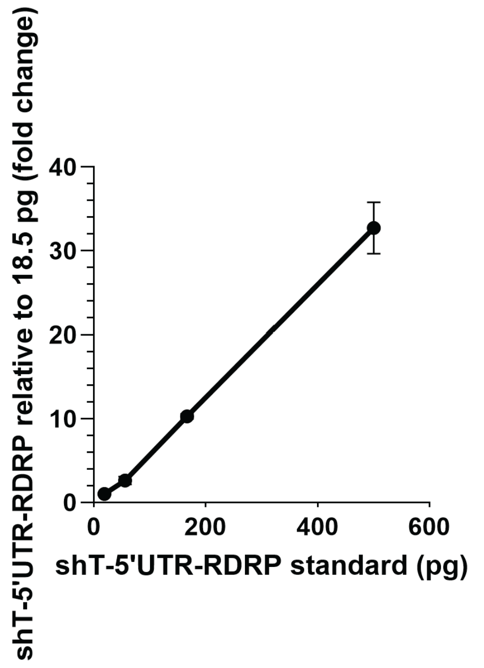

Fig. S3. Standard curve for quantification of shT-5′UTR-RDRP RNA by qRT-PCR. 1:3 serial dilutions of in vitro-synthesized shT-5′UTR-RDRP RNA, starting from 500 pg, were processed through the same RNA purification, reverse transcription, and quantitative PCR procedures used for ARMMs-derived RNA samples. qRT-PCR was performed using primers spanning the two shT-RNA modules and the intervening linker region. Relative RNA abundance (fold change) was calculated from Ct values, with the signal obtained from 18.5 pg of input RNA defined as 1. The resulting values were plotted against RNA input to generate the standard curve used to estimate the concentration of shT-5′UTR-RDRP RNA present in ARMMs preparations.

Supplemental Table 1

shRNA targets

**SUPT4H1**	GCAGCGAGTCAGTAACTTTAA
**SARS-Cov-2 RDRP**	
shRDRP-1	AGGAAGTTCTGTTGAATTAAA
shRDRP-2	TTACGATGGTGGCTGTATTAA
shRDRP-3	GGGTAAGGCTAGACTTTATTA
shRDRP-4	GCCTAACATGCTTAGAATTAT
shRDRP-5	GATAAGTATGTCCGCAATTTA
shRDRP-6	CCAACATGAAGAAACAATTTA
shRDRP-7	ACGTCAACGTCTTACTAAATA
**shRDRP-8**	CTGCATTGTGCAAACTTTAAT
shRDRP-9	GGAAGAAACAATTTATAAA
shRDRP-10	GCATATTTGCGTAAACATA
shRDRP-11	GTCTCTATCTGTAGTACTATG
shRDRP-12	CTACTATAACTCAAATGAATC
shRDRP-13	CTAGCATAAAGAACTTTAAGT
shRDRP-14	CCTAACATGCTTAGAATTATG
shRDRP-15	CTAACATGCTTAGAATTATGG
shRDRP-16	CAATGTTAATGCACTTTTATC
**SARS-Cov-2 5′UTR**	
sh5′UTR-1	AGTGCACTCACGCAGTATAAT
**sh5′UTR-2**	ACACGAGTAACTCGTCTATCTT
sh5′UTR-3	GGGTGTGACCGAAAGGTAAG
sh5′UTR-4	CGTTGACAGGACACGAGTAA
sh5′UTR-5	TGACAGGACACGAGTAACTC
sh5′UTR-6	AGATCTGTTCTCTAAACGAACT

Quantitative PCR primers

Taqman Primers

**SUPT4H1**	Primer-1	CTGACACCGCATATACACCTG
	Primer-2	CGAGAGATGGTATATGACTGCAC
	Probe	/56-FAM/CATCGCAAT /ZEN/GATTCCATCAAAGGAAGAGC/3IABkFQ/
**tomato GAPDH**	Primer-1	CTCTTTGGTGAGAAGGCTGTTA
	Primer-2	CGGTCGACTCCACAATGTAAT
	Probe	/5HEX/CCAGAGGAA /ZEN/ATTCCATGGGCACAGA/3IABkFQ/
**human GAPDH**	Primer-1	TGCACAATGCAGCTCTATAC TC
	Primer-2	CCAGGCTCAGATCTGGATAGA
	Probe	/5HEX/AAGGTCGGA /ZEN/GTCAACGGATTTGGTC/3IABkFQ/

SYBR green primers

**shT-5′UTR-RDRP**	Primer-1	TGCACAATGCAGCTCTATATCTC
	Primer-2	CCAGGCTCAGATCTGGATAGA
**shSUPT4H1-L**	Primer-1	GTGGAAAGGACGAAACACCGAGATCTGGGTCTCTCTGG
	Primer-2	AAAGTTACTGACTCGCTGCCCCGGATCGATAACAGATC
